# Ablação por Cateter para Fibrilação Atrial em Pacientes com Fração de Ejeção do Ventrículo Esquerdo ≤ 45%: Uma Metanálise de Ensaios Clínicos Randomizados

**DOI:** 10.36660/abc.20230214

**Published:** 2024-02-16

**Authors:** Yujie Cui, Jialu Yao, Junyi Zhang, Zhenghao Liu, Tan Chen, Yafeng Zhou

**Affiliations:** 1 Medical Center Soochow University Suzhou Dushu Lake Hospital Suzhou China Department of Cardiology, Dushu Lake Hospital Affiliated to Soochow University, Medical Center of Soochow University , Suzhou Dushu Lake Hospital , Suzhou – China; 2 Department of Cardiology The First Affiliated Hospital Soochow University Suzhou China Department of Cardiology , The First Affiliated Hospital of Soochow University , Suzhou – China

**Keywords:** Fibrilação Atrial, Insuficiência Cardíaca, Ablação por Cateter, Metanálise

## Abstract

**Fundamento:**

A fibrilação atrial (FA) e a insuficiência cardíaca (IC) coexistem frequentemente, resultando em desfechos adversos. No entanto, permanecem controvérsias quanto à eficácia da ablação por cateter (AC) em pacientes com FA com disfunção ventricular esquerda grave.

**Objetivos:**

O objetivo deste estudo foi realizar uma metanálise de ensaios prospectivos randomizados e controlados para avaliar a eficácia da AC versus terapia médica (TM) em pacientes com FA com fração de ejeção do ventrículo esquerdo (FEVE) ≤45%.

**Métodos:**

Procuramos na literatura estudos que comparassem AC com TM em pacientes com FA com FEVE ≤45%. Foi realizada uma metanálise de 7 ensaios clínicos, incluindo 1.163 pacientes com FA e IC. A análise de subgrupo foi realizada com base na FEVE basal. Todos os testes foram bilaterais; apenas o valor p <0,05 foi considerado estatisticamente significativo.

**Resultados:**

Descobrimos que a AC estava associada a menor mortalidade por todas as causas (taxa de risco: 0,52, IC 95%: 0,37 a 0,72; p<0,01) e maiores melhorias na FEVE (diferença média: 4,80%, IC 95%: 2,29% a 7,31%; p<0,01) em comparação com TM. Os pacientes do grupo AC apresentaram menor risco de hospitalização por IC e recorrência de FA e qualidade de vida significativamente melhor do que aqueles do grupo TM. Os resultados da análise de subgrupo indicaram que pacientes com disfunção ventricular esquerda mais leve melhoraram a FEVE após a ablação de FA (diferença média: 6,53%, IC 95%: 6,18% a 6,88%; p<0,01) em comparação com pacientes com doença mais grave (diferença média : 2,02%, IC 95%: 0,87% a 3,16%; p<0,01).

**Conclusões:**

Nossa metanálise demonstrou que a AC foi associada a melhorias significativas nos resultados de pacientes com FA com FEVE ≤45%. Além disso, pacientes com FA com disfunção ventricular esquerda mais leve poderiam se beneficiar mais com a AC.

## Introdução

Na prática clínica, a fibrilação atrial (FA) e a insuficiência cardíaca (IC) são condições cardíacas comuns. ^1 , 2^ Estas duas doenças coexistem frequentemente, resultando em desfechos clínicos adversos. ^3 , 4^ A ablação por cateter (AC) é uma estratégia terapêutica estabelecida para a FA. Evidências anteriores de ensaios clínicos randomizados (ECR) indicaram que a ablação de FA estava associada a resultados benéficos em pacientes com FA e IC. ^5 , 6^ Embora as diretrizes recomendem AC como uma opção de tratamento para certos pacientes selecionados com FA e IC, ^7^ não há um consenso claro sobre potenciais grupos de pacientes que poderiam se beneficiar da AC.

Recentemente, algumas metanálises descobriram que a AC melhora os resultados clínicos em pacientes com FA e IC, incluindo a função sistólica do ventrículo esquerdo e a mortalidade por todas as causas, em comparação com a terapia médica (TM). ^8 , 9^ No entanto, a função sistólica do ventrículo esquerdo dos pacientes variou entre os estudos incluídos nessas metanálises. O estudo CASTLE-AF inscreveu apenas pacientes com fração de ejeção do ventrículo esquerdo (FEVE) basal ≤35%. ^5^ Em contraste, o estudo RAFT-AF incluiu um subconjunto de pacientes com FEVE >45%. ^6^ Além disso, o estudo CABANA até inscreveu participantes com FEVE >50%. ^10^ Isso significou que alguns pacientes com disfunção diastólica ou sistólica leve foram incluídos nas metanálises publicadas. Dada a associação entre FEVE e mau prognóstico dos pacientes com IC, ^11^ os resultados dos estudos publicados podem ser influenciados, uma vez que alguns pacientes sem diminuição significativa da FEVE foram incluídos na análise. Esta metanálise de ECRs teve como objetivo explorar ainda mais o papel da ablação de FA em pacientes com IC com FEVE ≤45% em comparação com a terapia medicamentosa. Além disso, procuramos avaliar a correlação entre a FEVE média basal e a eficácia da AC, conduzindo uma metanálise de subgrupo pré-especificada comparando os ECRs incluídos no estudo.

## Métodos

Esta metanálise foi realizada de acordo com as recomendações descritas na declaração PRISMA (Preferred Reporting Items for Systematic Reviews and Meta-Analyses). ^12^

### Fontes de dados e estratégia de busca de estudos

Foram incluídos artigos sobre AC em pacientes com IC e FA publicados no PubMed, na Biblioteca Cochrane e na Web of Science até janeiro de 2023. Identificamos ECR usando os seguintes termos: “ *Atrial Fibrillation* ”, “ *Heart Failure* ” e “ *Radiofrequency Ablation* ”. Nossa pesquisa foi limitada a ensaios clínicos em humanos. Apenas a publicação em inglês com texto completo foi incluída. Também recuperamos referências cruzadas de artigos de revisão e diretrizes relevantes para identificar todos os estudos relevantes. Dois investigadores conduziram uma pesquisa de estudo de forma independente.

### Seleção de estudos

Apenas ECR prospectivos foram incluídos. Os critérios de inclusão foram os seguintes: 1) estudos clínicos incluíram pacientes com FA com FEVE ≤45%; 2) o grupo intervenção utilizou AC para controle do ritmo (o procedimento principal foi o isolamento das veias pulmonares); 3) o grupo controle utilizou TM apenas para tratamento; 4) os estudos relataram pelo menos um desfecho cardiovascular, como mortalidade por todas as causas, hospitalização por IC, FEVE e qualidade de vida.

## Resultados

Os desfechos clínicos para esta metanálise foram mortalidade por todas as causas, hospitalização por IC, recorrência de FA, melhora na FEVE e alterações na distância percorrida no teste de caminhada de 6 minutos (TC6) e nos escores do Minnesota Living with Heart Failure Questionnaire (MLHFQ).

### Extração de dados e avaliação de qualidade

Os dados foram obtidos por dois revisores consecutivos e relatados em formulários padronizados. Dois revisores extraíram características de cada estudo, incluindo características basais do paciente, duração do acompanhamento e desfechos de forma independente. A qualidade metodológica dos estudos incluídos foi avaliada usando a ferramenta Cochrane Risk of Bias. ^13^ O viés de publicação foi avaliado qualitativamente por gráfico de funil. Eventuais discrepâncias entre os revisores foram resolvidas pela discussão que chegou a um consenso.

### Análise de subgrupo

Os estudos incluídos foram divididos em dois grupos para metanálise de subgrupos com base no fato de a FEVE média basal ser superior a 30%. A subanálise pré-especificada foi realizada para testar se a eficácia da AC diferia de acordo com o subgrupo.

### Síntese de dados e análise estatística

Todas as análises foram avaliadas com base na intenção de tratar. Uma razão de risco (RR) com intervalo de confiança de 95% foi utilizada para estimar as variáveis categóricas, incluindo mortalidade por todas as causas, hospitalização por IC e recorrência de FA. A diferença média com IC 95% pesou as variáveis contínuas, incluindo alterações nos escores de FEVE, DTC6 e MLHFQ. A heterogeneidade entre os estudos foi avaliada com a estatística I ^2^ . A heterogeneidade foi considerada se o valor da estatística I ^2^ fosse >30%. As análises de sensibilidade foram realizadas removendo um ensaio individual de cada vez da metanálise para identificar a fonte de heterogeneidade. Todas as comparações foram bilaterais e quando valor de p <0,05, os resultados foram considerados estatisticamente significativos. Modelos de efeito aleatório foram utilizados para calcular todos os resultados agrupados. As análises estatísticas foram realizadas utilizando o pacote de software RevMan (Review Manager, Versão 5.4. Copenhagen, The Nordic Cochrane Centre, Cochrane Collaboration).

## Resultados

**Figura 1:**
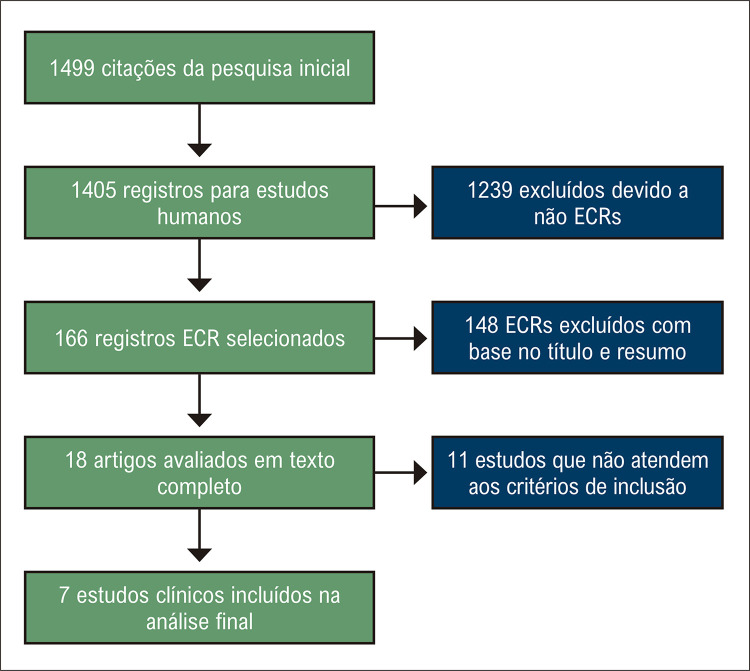
– Fluxograma de seleção dos ensaios clínicos.

Os resultados primários do nosso estudo estão resumidos na Figura Central . Procuramos na literatura estudos que comparassem AC com TM em pacientes com FA e IC. Nossa busca inicial resultou em 1.499 citações. A maioria desses artigos foi excluída por não serem ECRs. Após triagem com base no título e resumo, restaram 18 estudos para avaliação do texto completo. Destes, 11 artigos foram excluídos da análise final por não atenderem aos critérios de inclusão. Por fim, um total de 7 artigos foram incluídos na metanálise. ^5 6 , 14 - 18^ ( Figura 1 )

**Figura Central:**
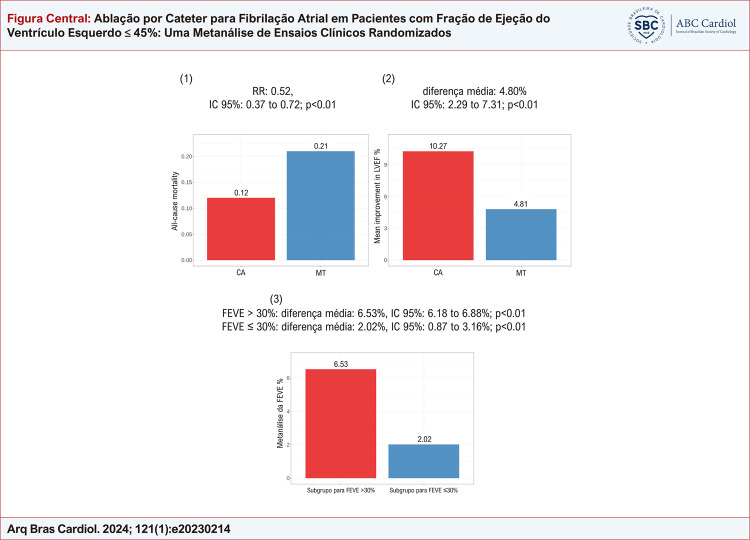
: Ablação por Cateter para Fibrilação Atrial em Pacientes com Fração de Ejeção do Ventrículo Esquerdo ≤ 45%: Uma Metanálise de Ensaios Clínicos Randomizados

(1) Mortalidade por todas as causas; (2) Melhora média da FEVE; (3) Análise de subgrupo para FEVE: AC: ablação por cateter; TM: terapia médica; FEVE: fração de ejeção do ventrículo esquerdo; IC: intervalo de confiança.

**Figura 2:**
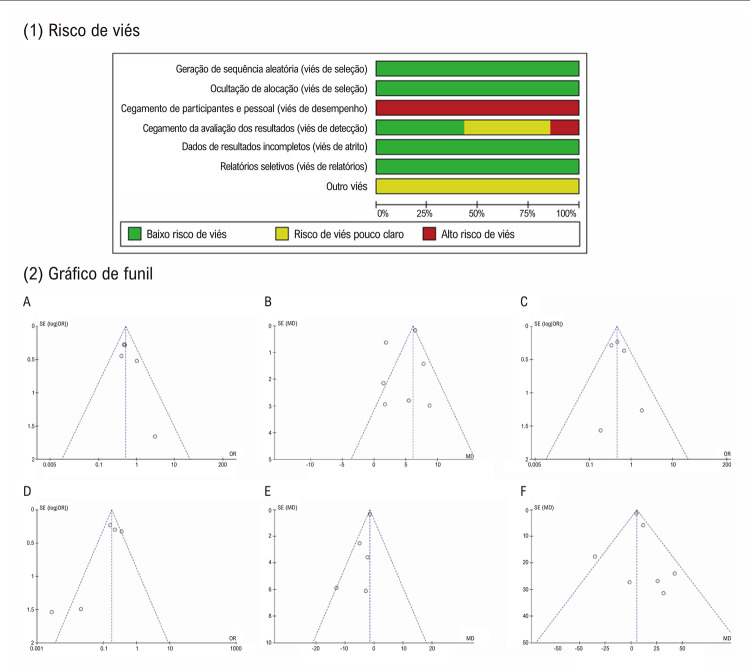
– 1) Risco de Viés; 2) Gráfico de funil. Gráficos de funil para (A) mortalidade por todas as causas, (B) melhora na FEVE, (C) hospitalização por IC, (D) recorrência de FA, (E) mudança nos escores do Minnesota Living with Heart Failure Questionnaire, (F) mudança em Teste de 6 minutos de caminhada. FEVE: fração de ejeção do ventrículo esquerdo, IC=insuficiência cardíaca, FA: fibrilação atrial.

As principais características dos ECRs que atenderam aos critérios de inclusão estão resumidas na Tabela 1 . Os 7 estudos clínicos incluíram 1.163 pacientes com FA com FEVE ≤45%. Os participantes de cada estudo foram alocados aleatoriamente na proporção de 1:1 para receber AC ou TM para FA. Quatro dos estudos incluídos geraram desenhos de randomização computadorizados usando randomização em blocos. ^6 , 16 - 18^ O estudo de MacDonald foi alocado por meio de uma sequência gerada por computador. ^14^ Nos estudos ARC-HF e CASTLE-AF, a randomização estratificada foi usada para garantir o equilíbrio de características iniciais. ^5 , 15^ No geral, 584 pacientes foram randomizados para o grupo de ablação de FA e 579 pacientes foram randomizados para o grupo de TM. A idade média dos pacientes nos estudos variou de 57 a 67 anos, 70% dos participantes eram do sexo masculino. Cinco ensaios incluídos inscreveram apenas pacientes com FA persistente, mas os ensaios CASTLE-AF e RAFT-AF também inscreveram pacientes com FA paroxística. No grupo AC dos estudos incluídos, o principal procedimento de ablação foi o isolamento das veias pulmonares. Ao mesmo tempo, nova ablação também foi realizada na maioria dos pacientes. Conforme mostrado na Figura 2 , a qualidade metodológica dos estudos incluídos foi classificada como de alta qualidade. Nenhum viés de publicação significativo foi observado nos gráficos de funil. No geral, houve um risco relativamente baixo de viés nesta análise.

**Tabela 1:** – Ensaios Clínicos Randomizados de AC Versus TM

Sigla de Estudo	MacDonald et al.	ARC-HF	AATAC	CÂMERA-RM	CASTELO-AF	AMICA	RAFT-AF
Ano	2011	2013	2016	2017	2018	2019	2022
Tamanho da amostra para ablação	22	26	102	33	179	98	124
Tamanho da amostra para TM	19	26	101	33	184	100	116
Idade Anos	63	63	61	61	64	65	67
Masc., %	78	87	74	91	86	90	74
Acompanhamento, Meses	6	12	24	6	60	12	24
NYHA Class	II/III	II-IV	II/III	II-IV	I-IV	II/III	II/III
Estratégia principal de ablação	IPV	IPV	IPV	IPV	IPV	IPV	IPV
Estratégia médica	Controle de taxa	Controle de taxa	Amiodarona	Controle de taxa	Controle de taxa ou ritmo	Controle de taxa ou ritmo	Controle de taxa
População de pacientes	FEVE<35%por RMC	FEVE≤35%por ventriculografia com radionuclídeos	FEVE≤40%por ecocardiografia transtorácica	FEVE≤45%por RMC	FEVE≤35% por ecocardiografia transtorácica	FEVE≤35%por ecocardiografia transtorácica	FEVE≤45%
FEVE no grupo de ablação	16,1 (7,1)	22 (8)	29(5)	32(9,4)	32,5(9,6)	27,8(9,5)	30,1(8,5)
FEVE no grupo médico	19,6 (5,5)	25 (7)	30(8)	34(7,8)	31,5(7,4)	24,8(8,8)	30,3(9,2)
FEVE média	17,7(6,5)	23,5(7,6)	29,5(6,6)	33(8,5)	32,2(7,9)	26,3(9,3)	30,2(8,7)

FA: fibrilação atrial; IC: insuficiência cardíaca; AC: ablação por cateter; TM: terapia médica; RM: ressonância magnética; NYHA: classe funcional da New York Heart Association; FEVE: fração de ejeção do ventrículo esquerdo; RMC: ressonância magnética cardiovascular; IVP: isolamento da veia pulmonar. Todos os ensaios incluídos adotaram um valor P bilateral de 0,05 para avaliar a significância estatística.

### Mortalidade por todas as causas

**Figura 3:**
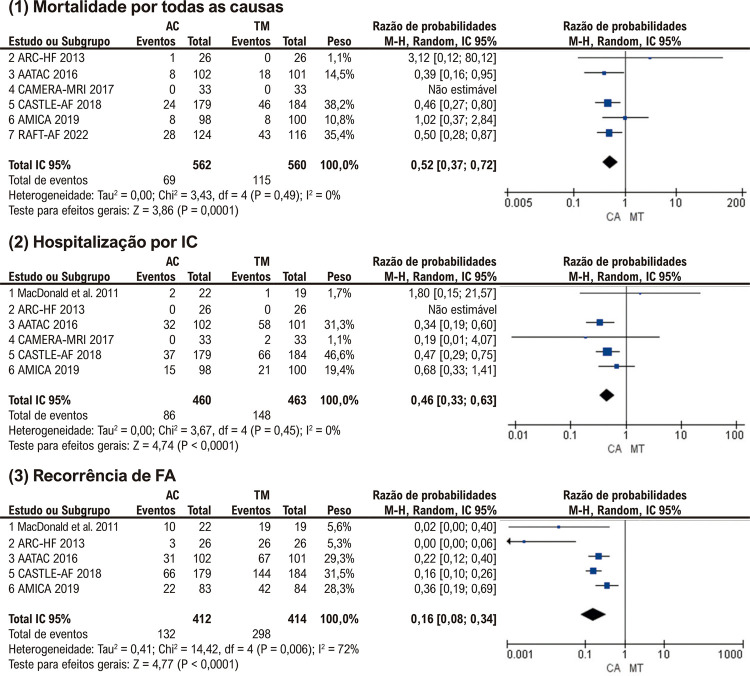
– Análises agrupadas para (1) Mortalidade por todas as causas, (2) hospitalização por IC, (3) recorrência de FA. AC: ablação por cateter; TM: terapia médica; IC: intervalo de confiança; HF: insuficiência cardíaca; FA: fibrilação atrial.

Seis estudos incluídos relataram dados sobre mortalidade por todas as causas no final do acompanhamento. Conforme exibido na Figura 3 (1), a AC foi associada a menor mortalidade por todas as causas em comparação com a TM (RR: 0,52; p<0,01). Não houve heterogeneidade entre estudos entre os ensaios.

### Melhoria na FEVE

**Figura 4:**
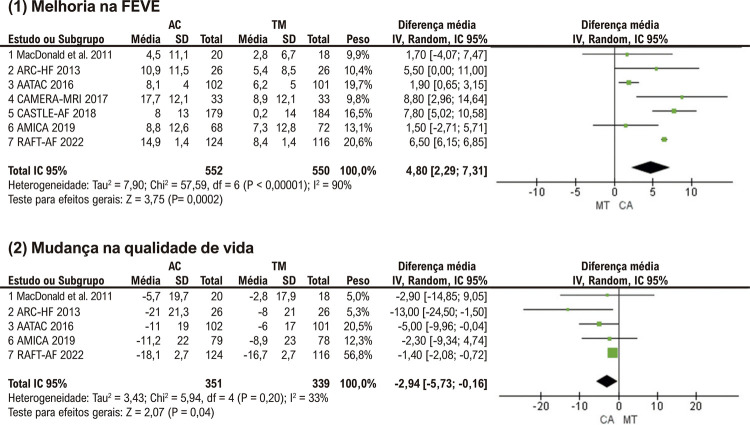
– Análises agrupadas para (1) Melhoria na FEVE, (2) Mudança na qualidade de vida. AC: ablação por cateter, TM: terapia médica; IC: intervalo de confiança; FEVE: fração de ejeção do ventrículo esquerdo.

Comparado ao grupo TM, o grupo AC foi associado a uma maior melhora na FEVE (MD: 4,80%; p<0,01) ( Figura 4 (1)). Notável heterogeneidade foi observada entre os ensaios. A análise de sensibilidade, excluindo um ensaio individual de cada vez, não reduziu significativamente a heterogeneidade. (Suplemento 1)

### Hospitalização por IC e recorrência de FA

Os dados de hospitalização por IC estavam disponíveis em 6 estudos. Comparado com o grupo TM, o grupo ablação teve uma taxa de hospitalização por IC reduzida (RR: 0,46; p<0,01) ( Figura 3 (2)). Nenhuma heterogeneidade significativa entre os estudos foi observada.

Cinco estudos relataram o número de pacientes que permaneceram com FA ao final do acompanhamento. Os resultados agrupados sugeriram que no grupo AC, a taxa de recorrência de FA dos pacientes foi significativamente menor do que no grupo TM (RR: 0,16; p<0,01) ( Figura 3 (3)). Foi detectada heterogeneidade significativa na análise, sensível à exclusão do ensaio ARC-HF (I2=52%). A taxa de recorrência de FA dos pacientes do grupo AC permaneceu menor após a exclusão do ARC-HF (RR: 0,21; p<0,01) (Suplemento 2).

### Mudança na qualidade de vida

Cinco estudos relataram dados sobre qualidade de vida com a pesquisa Minnesota Living with Heart Failure Questionnaire. Conforme mostrado na Figura 4 (2), o grupo de ablação de FA foi associado a uma diminuição mais significativa nos escores do MLHFQ do que o grupo TM (MD: -2,94 pontos; p=0,04), indicando uma maior melhora na qualidade de vida no grupo AC. Houve apenas uma leve heterogeneidade entre os ensaios.

### Melhoria na distância de caminhada de 6 minutos

**Figura 5:**
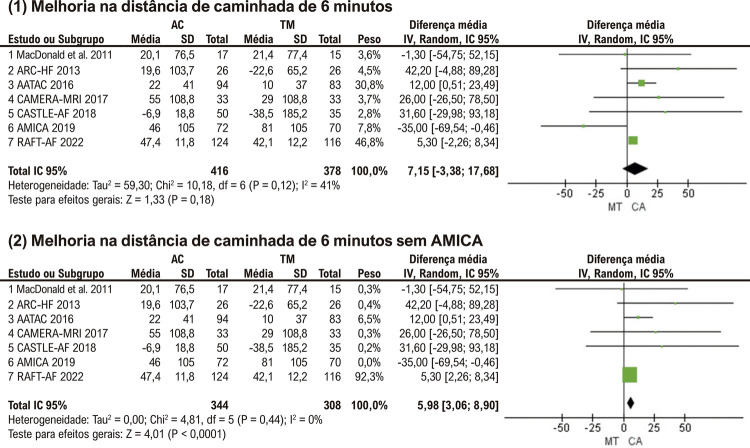
– Análises agrupadas para (1) Melhoria na distância de caminhada de 6 minutos; (2) Melhoria no Teste de 6 minutos de caminhada sem AMICA. AC: ablação por cateter; TM: terapia médica; IC: intervalo de confiança; AMICA: ablação por cateter versus melhor terapia médica em pacientes com fibrilação atrial persistente e insuficiência cardíaca congestiva: o estudo randomizado AMICA.

Os resultados de alteração na DTC6 estavam disponíveis em 7 estudos. A análise agrupada sugeriu não haver diferença significativa entre os grupos de ablação e médico (MD: 7,15 metros; p = 0,18) ( Figura 5 (1)). Heterogeneidade significativa foi observada entre os estudos e foi sensível à exclusão do estudo AMICA (I2=0%). Após a exclusão do ensaio, a análise mostrou um aumento significativo da DTC6 no grupo ablação em comparação ao grupo TM (MD: 5,98 metros; p<0,01) ( Figura 5 (2)).

### Análise de subgrupo

**Figura 6:**
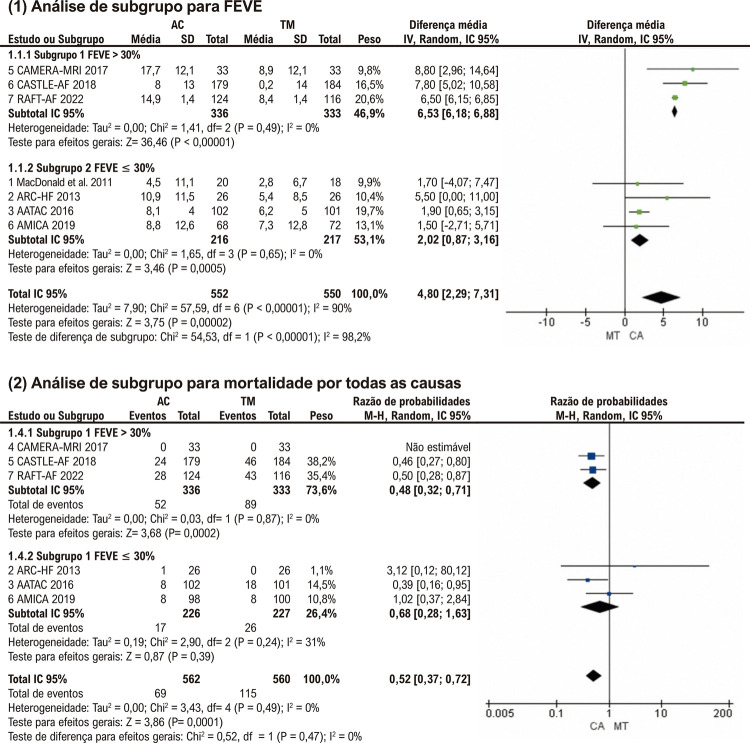
– Análise de subgrupo para (1) FEVE; (2) Mortalidade por todas as causas. AC: ablação por cateter, TM: terapia medicamentosa; IC: intervalo de confiança; FEVE: fração de ejeção do ventrículo esquerdo.

Realizamos uma análise de subgrupo de FEVE e mortalidade por todas as causas pela FEVE média basal ≤30% e >30%. Conforme mostrado na Figura 6 (1), em comparação com pacientes com FEVE média basal ≤30%, os pacientes com FEVE média basal >30% obtiveram uma melhora maior na FEVE com a ablação de FA (DM: 2,02%; p<0,01 vs. DM: 6,53%; p<0,01). A interação entre os subgrupos foi estatisticamente significativa (p<0,01) e não foi observada heterogeneidade entre estudos dentro de cada subgrupo. Além disso, descobrimos que para pacientes com FEVE média >30% no início do estudo, o risco de mortalidade por todas as causas foi menor no grupo AC do que no grupo TM (RR: 0,48; p<0,01), enquanto para pacientes com FEVE ≤30% , não houve diferença estatística na mortalidade por todas as causas entre os dois grupos (RR: 0,68; p=0,39) ( Figura 6 (2)). Embora tenha havido uma interação não estatisticamente significativa entre os subgrupos (p = 0,47), os resultados agrupados indicaram que os médicos deveriam considerar cuidadosamente se os pacientes com FA e IC poderiam se beneficiar da AC.

## Discussão

Os principais achados desta metanálise foram que a AC para FA em pacientes com FEVE ≤45% foi associada a reduções na mortalidade por todas as causas, hospitalização por IC e recorrência de FA em comparação com a TM. A ablação da FA também foi associada a melhorias mais significativas na FEVE e na qualidade de vida do que a terapia medicamentosa. Em suma, os pacientes com FA com FEVE ≤45% poderiam se beneficiar mais da AC em comparação com a TM. Além disso, os resultados das análises de subgrupo relataram que os pacientes com FEVE média basal >30% poderiam obter melhora da FEVE com a ablação de FA do que aqueles com FEVE ≤30%.

Sobre assuntos relacionados, diversas revisões sistemáticas e metanálises relataram melhor prognóstico clínico com a ablação de FA em pacientes com IC. ^9 , 19^ No entanto, a FEVE dos pacientes variou amplamente entre os estudos incluídos nessas análises. No estudo CABANA, mais de 70% dos pacientes apresentaram FEVE >50%; ^10^ enquanto o estudo AMICA incluiu pacientes com FEVE de 35% ou menos. ^18^ O nível de FEVE está intimamente correlacionado com o prognóstico de pacientes com IC. ^11^ Pacientes com FEVE mais elevada pode impactar os resultados da metanálise. Assim, é essencial realizar uma metanálise específica de ECRs que recrutem pacientes com FEVE mais baixa.

Identificamos 7 estudos que incluíram pacientes com FEVE ≤45% após uma pesquisa de artigos e realizamos uma análise agrupada subsequente deles. Em 2022, Şaylık et al. e Chang et al. publicaram novas metanálises sobre o papel da AC em pacientes com FA e IC, dos quais vários ECRs incluíram pacientes com FEVE >50%. ^9 , 19^ Em nosso estudo, excluímos o estudo CABANA que incluiu pacientes com FEVE >50%. ^10^ O estudo PABA-CHF também foi excluído porque o grupo controle foi submetido a ablação adicional do nó atrioventricular com estimulação biventricular em vez de TM. ^20^ O estudo RAFT-AF publicado recentemente realizou análises de subgrupos com base na FEVE basal dos pacientes. ^6^ Assim, diferentemente de Şaylık et al., incluímos a análise de subgrupo de RAFT-AF para pacientes com FEVE ≤45% em vez da análise completa. Acreditamos que nosso estudo possa beneficiar os médicos na avaliação de pacientes com FA com menor FEVE e com menor candidatura para AC.

Pacientes com FA com IC tendem a ter maior mortalidade e resultados ruins. ^3^ Estudos anteriores sugerem que a FA pode causar a deterioração constante da disfunção ventricular esquerda. ^21^ O controle do ritmo e a manutenção do ritmo sinusal são as chaves para o tratamento clínico em pacientes com FA com IC. ^22^ Cateter a ablação é uma estratégia terapêutica estabelecida para controle do ritmo em pacientes com FA. Nossa análise conjunta descobriu que, para pacientes com FEVE ≤45%, a AC para FA foi associada a melhores resultados clínicos, incluindo menor mortalidade por todas as causas e taxa de hospitalização por IC, além de maior FEVE. Conforme relatado em nosso estudo, a ablação da FA também poderia melhorar significativamente a qualidade de vida do paciente e reduzir a carga da doença. Portanto, os médicos devem levar em consideração os benefícios da AC ao tomar a decisão clínica para pacientes com FA com FEVE ≤45%.

A distância percorrida no teste de caminhada de seis minutos é um preditor independente de prognóstico de IC. ^23^ Em pacientes com IC, a DTC6 está intimamente associada à mortalidade por todas as causas e à qualidade de vida. ^24^ A análise conjunta da distância percorrida no teste de caminhada de seis minutos não mostrou diferença significativa entre a distância percorrida no teste de ablação e grupo médico. Esta análise teve heterogeneidade significativa entre estudos e foi sensível à exclusão do AMICA. ^18^ Após excluir o AMICA, descobrimos que o grupo AC teve uma melhora mais significativa na DTC6 do que o grupo TM. Os investigadores do ensaio AMICA encerraram o estudo precocemente, o que pode ser a causa da heterogeneidade. A confiabilidade e a estabilidade da nossa análise agrupada são limitadas devido à heterogeneidade significativa. O efeito da AC na DTC6 necessita de maior exploração em estudos futuros.

Além disso, notamos diferença no grau de melhora da FEVE no grupo AC em comparação ao grupo TM entre os estudos incluídos na metanálise. Nos ensaios CAMERA-MRI, ^17^ CASTLE-AF ^5^ e RAFT-AF, ^6^ a melhora da FEVE no grupo de ablação aumentou em mais de 6% em comparação ao grupo de medicamentos (8,80% no CAMERA-MRI, 7,80% no CASTLE-AF, 6,50% em RAFT-AF). No entanto, os outros quatro estudos tiveram melhorias relativamente menores na FEVE (1,70% no MacDonald’s, ^14^ 5,50% no ARC-HF, ^15^ 1,90% no AATAC ^16^ e 1,50% no AMICA ^18^ ). Nos ensaios MacDonald’s, AATAC e AMICA, a melhoria da FEVE no grupo de ablação foi ainda inferior a 2%. Ao comparar esses estudos incluídos, descobrimos que a FEVE média basal foi superior a 30% nos ensaios CAMERA-MRI, CASTLE-AF e RAFT-AF, enquanto a FEVE foi inferior a 30% nos outros quatro estudos. ( Tabela 1 ) Para explorar isso ainda mais, realizamos análises de subgrupos de FEVE e mortalidade por todas as causas pela FEVE média basal ≤30% e >30%. Os resultados das análises de subgrupos relataram que a melhora da FEVE foi mais significativa em pacientes com FEVE >30% do que naqueles com FEVE ≤30%. Além disso, nenhuma diferença na mortalidade por todas as causas entre os grupos AC e TM foi observada na metanálise de pacientes incluídos no subgrupo com FEVE média basal ≤30%. A subanálise do estudo CASTLE-AF observou resultados semelhantes, onde pacientes com FEVE basal grave (<20%) no grupo AC apresentaram desfechos adversos com mais frequência em comparação com pacientes com FEVE basal moderada (≥20% e <35%). ^25^ Em uma palavra, apesar da grosseria dos nossos critérios de agrupamento, esses achados indicaram que a AC para FA em pacientes com melhor FEVE foi associada a maiores melhorias nos desfechos do que aqueles com disfunção ventricular esquerda mais grave. A FEVE de 30% pode ser um critério de estratificação para os médicos avaliarem a candidatura do paciente com IC à AC.

Além disso, descobrimos que algumas outras características basais dos pacientes também podem estar relacionadas aos resultados clínicos do estudo. Os resultados do estudo de MacDonald ^14^ não relataram diferença na hospitalização por IC entre os grupos AC e TM. No estudo ARC-HF, ^15^ a mortalidade por todas as causas foi maior no grupo de ablação de FA do que no grupo de TM. Em comparação com os outros ensaios, ^6 , 16 , 17^ a duração da FA nos pacientes dos ensaios MacDonald’s e ARC-HF foi significativamente mais longa, ultrapassando os 40 meses. Sugeriu que pacientes com duração prolongada de FA podem ter levado a um menor benefício da AC. Assim, a AC precoce para FA em pacientes com função sistólica prejudicada pode ser crucial para melhorar os resultados clínicos. A maioria dos participantes nos ensaios incluídos apresentava FA persistente, ^14 - 18^ enquanto os ensaios CASTLE-AF ^5^ e RAFT-AF ^6^ inscreveram um subconjunto de pacientes com FA paroxística. Em ambos os ensaios, o aumento da FEVE associado ao grupo de ablação foi maior do que nos outros ensaios que incluíram apenas pacientes com IC persistente. Isto indicou que os pacientes com IC e FA paroxística podem ser melhores candidatos à ablação. No entanto, o ensaio AATAC, ^16^ que incluiu pacientes com IC e FA persistente, descreveu a redução mais significativa na mortalidade por todas as causas associada à ablação entre os ensaios incluídos. Uma possível explicação dos resultados poderia ser que o grupo TM no estudo AATAC foi tratado com amiodarona para controlar o ritmo. Os efeitos colaterais da amiodarona podem aumentar os resultados adversos no grupo de medicamentos.

Além disso, observamos também que o diâmetro do átrio esquerdo dos pacientes do grupo de ablação dos estudos ARC-HF ^15^ e AMICA ^18^ foi maior que nos demais estudos, ^5 , 6 , 16 , 17^ chegando a 50 mm. Os resultados agrupados de ambos os ensaios relataram que a mortalidade por todas as causas no grupo AC de ambos os ensaios foi maior do que no grupo TM. Um diâmetro atrial esquerdo maior pode estar associado a disfunção cardíaca mais grave e maior carga de FA. Sugeriu ainda que pacientes com IC grave poderiam se beneficiar menos com a ablação de FA. Em resumo, os efeitos da ablação da FA variam em grupos de pacientes com IC com características diferentes, portanto a seleção de pacientes com IC a serem submetidos à AC deve ser considerada com cautela. São necessários mais ensaios clínicos controlados para explorar os critérios de estratificação e identificar potenciais grupos de pacientes com IC que poderiam beneficiar mais da ablação da FA.

### Limitações do estudo

Primeiro, este estudo atual foi limitado por critérios de elegibilidade inconsistentes para cada ensaio. As diferenças podem resultar em heterogeneidade ao combinar os dados destes ensaios. Em segundo lugar, o ensaio RAFT-AF forneceu ao desfecho primário um desfecho composto, que pode impactar os resultados da análise. Além disso, a análise de subgrupo baseou-se na FEVE média basal dos pacientes em cada estudo. A FEVE média não conseguiu representar a função ventricular esquerda de cada paciente nos estudos.

No entanto, nosso trabalho forneceu novas ideias para estudos futuros. Além disso, devido aos problemas do próprio estudo, os médicos e os pacientes não ficaram cegos ao processo de tratamento. A vontade subjetiva de médicos e pacientes pode afetar os resultados.

## Conclusões

Até onde sabemos, esta é a primeira vez que avaliamos a eficácia da AC em pacientes com FA com FEVE ≤45% em qualquer uma das metanálises publicadas até o momento. Nosso estudo demonstrou que, em comparação com a TM, a AC para FA em pacientes com IC foi associada a um menor risco de mortalidade por todas as causas, hospitalização por IC e recorrência de FA, além de melhorias mais significativas na FEVE e na qualidade de vida. Além disso, a subanálise revelou que os pacientes com disfunção ventricular esquerda mais leve poderiam se beneficiar mais da ablação da FA do que os pacientes com doença mais grave. Mais pesquisas são necessárias para explorar as características dos pacientes com FA e IC que poderiam se beneficiar da AC.
