# Supplementary material for: Ablação por Cateter para Fibrilação Atrial em Pacientes com Fração de Ejeção do Ventrículo Esquerdo ≤ 45%: Uma Metanálise de Ensaios Clínicos Randomizados
Source: Arq Bras Cardiol. 2024 Feb 16;121(1):e20230214. [Article in Portuguese] doi: 10.36660/abc.20230214 (PMC11098583; doi:10.36660/abc.20230214)
Supplement: Translation

# Translation: Catheter Ablation for Atrial Fibrillation in Patients with Left Ventricular Ejection Fraction ≤ 45%: A Meta-Analysis of Randomized Controlled Trials

**Authors:** Yujie Cui, Jialu Yao, Junyi Zhang, Zhenghao Liu, Tan Chen, Yafeng Zhou

**Affiliations:** 1 China Department of Cardiology, Dushu Lake Hospital Affiliated to Soochow University, Medical Center of Soochow University, Suzhou Dushu Lake Hospital, Suzhou – China; 2 China Department of Cardiology, The First Affiliated Hospital of Soochow University, Suzhou – China

**Keywords:** Atrial Fibrillation, Heart Failure, Catheter Ablation, Meta-Analysis

## Abstract

**Background:**

Atrial fibrillation (AF) and heart failure (HF) frequently coexist, resulting in adverse outcomes. However, controversies remain regarding the efficacy of catheter ablation (CA) in AF patients with severe left ventricular dysfunction.

**Objectives:**

The purpose of this study was to perform a meta-analysis of prospective randomized controlled trials to evaluate the efficacy of CA versus medical therapy (MT) in AF patients with left ventricular ejection fraction (LVEF) ≤45%.

**Methods:**

We searched the literature for studies that compared CA to MT in AF patients with LVEF ≤45%. A meta-analysis of 7 clinical trials was performed, including 1163 patients with AF and HF. Subgroup analysis was performed based on baseline LVEF. All tests were 2-sided; only the p-value <0.05 was considered statistically significant.

**Results:**

We found that CA was associated with lower all-cause mortality (risk ratio: 0.52, 95% CI: 0.37 to 0.72; p<0.01) and greater improvements in LVEF (mean difference: 4.80%, 95% CI: 2.29% to 7.31%; p<0.01) compared to MT. Patients in the CA group had a lower risk of HF hospitalization and AF recurrence and a significantly better quality of life than those in the MT group. The results of subgroup analysis indicated that patients with milder left ventricular dysfunction improved LVEF after AF ablation (mean difference: 6.53%, 95% CI: 6.18% to 6.88%; p<0.01) compared to patients with more severe disease (mean difference: 2.02%, 95% CI: 0.87% to 3.16%; p<0.01).

**Conclusions:**

Our meta-analysis demonstrated that CA was associated with significant improvements in outcomes of AF patients with LVEF ≤45%. Additionally, AF patients with milder left ventricular dysfunction could benefit more from CA.

## Introduction

In clinical practice, atrial fibrillation (AF) and heart failure (HF) are common cardiac conditions. ^1 , 2^ These two diseases frequently coexist, resulting in adverse clinical outcomes. ^3 , 4^ Catheter ablation (CA) is an established therapeutic strategy for AF. Previous evidence from randomized controlled trials (RCTs) indicated that AF ablation was associated with beneficial outcomes in AF patients with HF. ^5 , 6^ Although guidelines recommend CA as a treatment option for certain selected patients with AF and HF, ^7^ there is no clear consensus on potential patient groups that could benefit from CA.

Recently, some meta-analyses have found that CA improves clinical outcomes in AF patients with HF, including left ventricular systolic function and all-cause mortality, compared with medical therapy (MT). ^8 , 9^ However, patients’ left ventricular systolic function varied among the studies included in these meta-analyses. The CASTLE-AF trial enrolled only patients with baseline left ventricular ejection fraction (LVEF) ≤35%. ^5^ In contrast, the RAFT-AF trial included a subset of patients with LVEF >45%. ^6^ Additionally, the CABANA trial even enrolled participants with LVEF >50%. ^10^ This meant that some diastolic or mild systolic dysfunction patients were included in the published meta-analyses. Given the association between LVEF and poor prognosis of HF patients, ^11^ the results of the published studies may be influenced as some patients with no significant decrease in LVEF were included in the analysis. This meta-analysis of RCTs was aimed to further explore the role of AF ablation in HF patients with LVEF ≤45% compared to drug therapy. Additionally, we sought to evaluate the correlation between baseline mean LVEF and the efficacy of CA by conducting a prespecified subgroup meta-analysis comparing RCTs included in the study.

## Methods

This meta-analysis was performed according to the recommendations described in the PRISMA (Preferred Reporting Items for Systematic Reviews and Meta-Analyses) statement. ^12^

### Data sources and study search strategy

Papers regarding CA in patients with HF and AF that were published on PubMed, the Cochrane Library, and the Web of Science until January 2023 were included. We identified RCTs using the following terms: “Atrial Fibrillation,” “Heart Failure,” and “Radiofrequency Ablation.” Our search was limited to clinical trials in humans. Only full-text English publication was included. We also retrieved cross-references of relevant review articles and guidelines to identify all relevant studies. Two investigators conducted a study search independently.

### Study selection

Only prospective randomized clinical trials were included. The inclusion criteria were as follows: 1) clinical studies enrolled AF patients with LVEF ≤45%; 2) the intervention group used CA to control rhythm (the principal procedure was pulmonary vein isolation); 3) the control group used MT for treatment only; 4) the studies reported at least one cardiovascular endpoint, such as all-cause mortality, HF hospitalization, LVEF and quality of life.

### Outcomes

The clinical outcomes for this meta-analysis were all-cause mortality, HF hospitalization, AF recurrence, improvements in LVEF, and changes in 6-minute walk distance (6MWD) and Minnesota Living with Heart Failure Questionnaire (MLHFQ) scores.

### Data extraction and quality assessment

Data were obtained by 2 reviewers back-to-back and reported on standardized forms. Two reviewers extracted characteristics of each study, including baseline patient characteristics, follow-up duration, and end points independently. The methodological quality of the included studies was assessed using the Cochrane Risk of Bias Tool. ^13^ Publication bias was assessed qualitatively by funnel plot. Any discrepancies between the reviewers were resolved by the discussion that arrived at a consensus.

### Subgroup analysis

The included studies were divided into two groups for subgroup meta-analysis based on whether the baseline mean LVEF was greater than 30%. The prespecified sub-analysis was conducted to test whether the efficacy of CA differed according to subgroup.

### Data synthesis and statistical analysis

All analyses were assessed on an intention-to-treat basis. A risk ratio (RR) with a 95% confidence interval (CI) was used to estimate the categorical variables, including all-cause mortality, hospitalization for HF, and AF recurrence. Mean difference (MD) with 95% CI weighed the continuous variables, including changes in LVEF, 6MWD, and MLHFQ scores. Heterogeneity among studies was assessed with the I ^2^ statistic. Heterogeneity was considered if the value of the I ^2^ statistic was >30%. Sensitivity analyses were conducted by removing one individual trial at a time from the meta-analysis to identify the source of heterogeneity. All comparisons were 2-sided, and when a p-value <0.05, the results were considered statistically significant. Random-effect models were used to calculate all pooled results. Statistical analyses were performed using the RevMan software package (Review Manager, Version 5.4. Copenhagen, The Nordic Cochrane Centre, the Cochrane Collaboration).

## Results

The primary results of our study are summarized in the Central Illustration . We searched the literature for studies that compared CA to MT in patients with AF and HF. Our initial search resulted in 1499 citations. Most of these articles were excluded due to non-RCTs. After being screened based on title and abstract, 18 studies remained for full-text evaluation. Of these, 11 articles were excluded from the final analysis because they did not meet the inclusion criteria. Finally, a total of 7 articles were included in the meta-analysis. ^5 , 6 , 14 - 18^ ( Figure 1 )

**Central Illustration:** : Catheter Ablation for Atrial Fibrillation in Patients with Left Ventricular Ejection Fraction ≤ 45%: A Meta-Analysis of Randomized Controlled Trials

(1) All-cause mortality; (2) Mean improvement in LVEF; (3) Subgroup analysis for LVEF. CA: catheter ablation; MT: medical therapy; LVEF: left ventricular ejection fraction; CI: confidence interval.

**Figure 1:** – Flowchart of Clinical Trials Selection.

The main characteristics of the RCTs that met the inclusion criteria are summarized in Table 1 . The 7 clinical studies included 1163 AF patients with LVEF ≤45%. The participants in each study were randomly allocated in a 1:1 ratio to receive CA or MT for AF. Four of the included studies generated computerized randomization designs using block randomization. ^6 , 16 - 18^ MacDonald’s study was allocated through a computer-generated sequence. ^14^ In the ARC-HF and CASTLE-AF trials, stratified randomization was used to ensure the balance of baseline characteristics. ^5 , 15^ Overall, 584 patients were randomly assigned to the AF ablation group, and 579 patients were randomized to the MT group. The mean age of patients in the studies ranged from 57 to 67 years, 70% of the participants were males. Five included trials enrolled patients with persistent AF only, but the CASTLE-AF and RAFT-AF trials also enrolled patients with paroxysmal AF. In the CA group of the included trials, the key ablation procedure was pulmonary vein isolation. At the same time, further ablation was also performed in most patients. As shown in Figure 2 , the methodological quality of the included studies was classified as high-quality. No significant publication bias was observed in funnel plots. Overall, there was a relatively low risk of bias in this analysis.

**Table 1:** – Randomized Clinical Trials of CA Versus MT

Study Acronym	MacDonald et al.	ARC-HF	AATAC	CAMERA-MRI	CASTLE-AF	AMICA	RAFT-AF
Year	2011	2013	2016	2017	2018	2019	2022
Sample size for ablation	22	26	102	33	179	98	124
Sample size for MT	19	26	101	33	184	100	116
Age, years	63	63	61	61	64	65	67
Male, %	78	87	74	91	86	90	74
Follow-up, Months	6	12	24	6	60	12	24
NYHA class	II/III	II-IV	II/III	II-IV	I-IV	II/III	II/III
Principal ablation strategy	PVI	PVI	PVI	PVI	PVI	PVI	PVI
Medical strategy	Rate control	Rate control	Amiodarone	Rate control	Rate or rhythm control	Rate or rhythm control	Rate control
Patients population	LVEF<35% by CMR	LVEF≤35% by radionuclide ventriculography	LVEF≤40% by transthoracic echocardiography	LVEF ≤45% by CMR	LVEF≤35% by transthoracic echocardiography	LVEF≤35% by transthoracic echocardiography	LVEF≤45%
LVEF in the ablation group	16.1 (7.1)	22 (8)	29(5)	32(9.4)	32.5(9.6)	27.8(9.5)	30.1(8.5)
LVEF in the medical group	19.6 (5.5)	25 (7)	30(8)	34(7.8)	31.5(7.4)	24.8(8.8)	30.3(9.2)
Average LVEF	17.7(6.5)	23.5(7.6)	29.5(6.6)	33(8.5)	32.2(7.9)	26.3(9.3)	30.2(8.7)

AF: atrial fibrillation; HF: heart failure; CA: catheter ablation; MT: medical therapy; MRI: magnetic resonance imaging; NYHA: New York Heart Association functional class; LVEF: left ventricular ejection fraction; CMR: cardiovascular magnetic resonance; PVI: pulmonary vein isolation. All included trials adopted a 2-sided P-value of 0.05 to evaluate statistical significance.

**Figure 2:** – (1) Risk of Bias; (2) Funnel plot. Funnel plots for (A) All-cause mortality, (B) Improvement in LVEF, (C) HF hospitalization, (D) AF recurrence, (E) change in Minnesota Living with Heart Failure Questionnaire scores, (F) change in 6 Minute Walk Distance. LVEF: left ventricular ejection fraction; HF: heart failure; AF: atrial fibrillation.

### All-cause mortality

Six included studies reported data on all-cause mortality at the end of follow-up. As displayed in Figure 3 (1), CA was associated with lower all-cause mortality compared with MT (RR: 0.52; p<0.01). There was no interstudy heterogeneity among trials.

**Figure 3:** – Pooled analyses for (1) All-cause mortality, (2) HF hospitalization, (3) AF recurrence. CA: catheter ablation; MT: medical therapy; CI: confidence interval; HF: heart failure; AF: atrial fibrillation.

### Improvement in LVEF

Compared with the MT group, the CA group was associated with a greater improvement in LVEF (MD: 4.80%; p<0.01) ( Figure 4 (1)). Remarkable heterogeneity was observed among trials. Sensitivity analysis by excluding one individual trial at a time did not significantly reduce the heterogeneity. (Supplementary 1)

**Figure 4:** – Pooled analyses for (1) Improvement in LVEF, (2) Change in quality of life. CA: catheter ablation; MT: medical therapy; CI: confidence interval; LVEF: left ventricular ejection fraction.

### HF hospitalization and AF recurrence

HF hospitalization data were available in 6 studies. Compared with the MT group, the ablation group had a reduced HF hospitalization rate (RR: 0.46; p<0.01) ( Figure 3 (2)). No significant heterogeneity among studies was observed.

Five studies reported the number of patients who remained with AF at the end of follow-up. The pooled results suggested that in the CA group, the AF recurrence rate of patients was significantly lower than those in the MT group (RR: 0.16; p<0.01) ( Figure 3 (3)). Significant heterogeneity was detected in the analysis, which was sensitive to the exclusion of the ARC-HF trial (I ^2^ =52%). The AF recurrence rate of patients in the CA group remained lower after the exclusion of ARC-HF (RR: 0.21; p<0.01) (Supplementary 2).

### Change in quality of life

Five studies reported data on quality of life with the MLHFQ survey. As shown in Figure 4 (2), the AF ablation group was associated with a more significant decrease in MLHFQ scores than the MT group (MD: -2.94 points; p=0.04), indicating a greater improvement in quality of life in the CA group. There was just mild heterogeneity among trials.

### Improvement in 6-Minute Walk Distance

The results of change in 6MWD were available in 7 studies. The pooled analysis suggested no significant difference between ablation and medical groups (MD: 7.15 meters; p=0.18) ( Figure 5 (1)). Significant heterogeneity was observed among studies, and it was sensitive to the exclusion of the AMICA trial (I ^2^ =0%). After the exclusion of the trial, the analysis showed a significant increase of 6MWD in the ablation group compared to the MT group (MD: 5.98 meters; p<0.01) ( Figure 5 (2)).

**Figure 5:** – Pooled analyses for (1) Improvement in 6-Minute Walk Distance; (2) Improvement in 6-Minute Walk Distance without AMICA. CA: catheter ablation; MT: medical therapy; CI: confidence interval; AMICA: Catheter Ablation Versus Best Medical Therapy in Patients With Persistent Atrial Fibrillation and Congestive Heart Failure: The Randomized AMICA Trial.

### Subgroup analysis

We performed a subgroup analysis of LVEF and all-cause mortality by baseline average LVEF ≤30% and >30%. As shown in Figure 6 (1), compared to patients with baseline mean LVEF ≤30%, patients with baseline mean LVEF >30% got a greater improvement in LVEF from AF ablation (MD: 2.02%; p<0.01 vs. MD: 6.53%; p<0.01). The interaction between subgroups was statistically significant (p<0.01), and no interstudy heterogeneity was observed within each subgroup. Additionally, we found that for patients with average LVEF >30% at baseline, the risk of all-cause mortality was lower in the CA group than MT group (RR: 0.48; p<0.01), while for patients with LVEF ≤30%, there was no statistical difference in all-cause mortality between the two groups (RR: 0.68; p=0.39) ( Figure 6 (2)). Although there was a non-statistically significant interaction between subgroups (p=0.47), the pooled results indicated that clinicians should consider carefully whether AF patients with HF could benefit from CA.

**Figure 6:** – Subgroup analysis for (1) LVEF; (2) All-cause mortality. CA: catheter ablation; MT: medical therapy; CI: confidence interval; LVEF: left ventricular ejection fraction.

## Discussion

The main findings of this meta-analysis were that CA for AF in patients with LVEF ≤45% was associated with reductions in all-cause mortality, HF hospitalization, and AF recurrence compared to MT. AF ablation was also associated with more significant improvements in LVEF and quality of life than drug therapy. In a word, AF patients with LVEF ≤45% could benefit more from CA in comparison with MT. In addition, the results of the subgroup analyses reported that patients with baseline mean LVEF >30% could get LVEF improvement from AF ablation than those with LVEF ≤30%.

On related subjects, several systematic reviews and meta-analyses reported better clinical prognosis with AF ablation in HF patients. ^9 , 19^ However, the LVEF of patients varied widely among the studies included in these analyses. In the CABANA trial, more than 70% of patients had LVEF >50%; ^10^ while the AMICA trial included patients with LVEF of 35% or less. ^18^ The level of LVEF is closely correlated with the prognosis of HF patients. ^11^ Patients with higher LVEF may impact the results of the meta-analysis. Thus, conducting a specific meta-analysis of RCTs recruiting patients with lower LVEF is essential.

We identified 7 studies that enrolled patients with LVEF ≤45% after a search of articles and performed a subsequent pooled analysis of them. In 2022, Şaylık et al. and Chang et al. published new meta-analyses on the role of CA in AF patients with HF, of which several RCTs comprised patients with LVEF >50%. ^9 , 19^ In our study, we excluded the CABANA trial enrolling patients with LVEF >50%. ^10^ The PABA-CHF trial was also excluded because the control group underwent additional atrioventricular-node ablation with biventricular pacing instead of MT. ^20^ The recently published RAFT-AF trial performed subgroup analyses based on the baseline LVEF of patients. ^6^ So, unlike Şaylık et al. work, we included the subgroup analysis of RAFT-AF for patients with LVEF ≤45% instead of the complete analysis. We believe our study could benefit clinicians when assessing AF patients with lower LVEF candidacy for CA.

AF patients with HF tend to have higher mortality and poor outcomes. ^3^ Previous studies suggest AF could cause the steady deterioration of left ventricular dysfunction. ^21^ Rhythm control and maintenance of sinus rhythm are the keys to clinical treatment in AF patients with HF. ^22^ CA is an established therapeutic strategy for rhythm control in patients with AF. Our pooled analysis found that for patients with LVEF ≤45%, the CA for AF was associated with improved clinical outcomes, including lower all-cause mortality and HF hospitalization rate and higher LVEF. As reported in our study, ablation of AF could also significantly improve patient’s quality of life and reduce their disease burden. Hence, clinicians should take complete account of the benefits of CA when making the clinical decision for AF patients with LVEF ≤45%.

Six-minute walk distance is an independent predictor of HF prognosis. ^23^ In HF patients, 6MWD is closely associated with all-cause mortality and quality of life. ^24^ The pooled analysis regarding the 6-minute walk distance showed no significant difference between the ablation and medical group. This analysis had significant interstudy heterogeneity and was sensitive to the exclusion of AMICA. ^18^ After excluding AMICA, we found that the CA group had a more significant improvement in 6MWD than the MT group. The investigators of the AMICA trial terminated the study early, which might be the cause of the heterogeneity. The reliability and stability of our pooled analysis are limited due to significant heterogeneity. The effect of CA in 6MWD needed further exploration in future studies.

Furthermore, we noticed a difference in the degree of LVEF improvement in the CA group compared to the MT group among the studies included in the meta-analysis. In the CAMERA-MRI, ^17^ CASTLE-AF ^5^ and RAFT-AF ^6^ trials, LVEF improvement in the ablation group was increased by more than 6% compared to the drug group (8.80% in CAMERA-MRI, 7.80% in CASTLE-AF, 6.50% in RAFT-AF). However, the other four studies had relatively lower improvements in LVEF (1.70% in MacDonald’s, ^14^ 5.50% in ARC-HF, ^15^ 1.90% in AATAC, ^16^ and 1.50% in AMICA ^18^ ). In the MacDonald’s, AATAC, and AMICA trials, the improvement of LVEF in the ablation group was even less than 2%. By comparing these included studies, we found that the baseline mean LVEF was greater than 30% in the CAMERA-MRI, CASTLE-AF, and RAFT-AF trials, while the LVEF was less than 30% in the other four studies. ( Table 1 ) To explore this further, we performed subgroup analyses of LVEF and all-cause mortality by baseline average LVEF ≤30% and >30%. Results of the subgroup analyses reported that the LVEF improvement was more significant in patients with LVEF >30% than those with LVEF ≤30%. In addition, no difference in all-cause mortality between the CA and MT groups was observed in the meta-analysis of subgroup enrolling patients with baseline mean LVEF ≤30%. The sub-analysis of the CASTLE-AF trial noted similar findings, where patients with severe baseline LVEF (<20%) in the CA group experienced adverse endpoints more often compared to patients with moderate baseline LVEF (≥20% and <35%). ^25^ In a word, despite the crudeness of our grouping criteria, these findings indicated that CA for AF in patients with a better LVEF was associated with greater improvements in outcomes than those with more severe left ventricular dysfunction. LVEF of 30% might be a stratification criterion for clinicians to assess the HF patient’s candidacy for CA.

In addition, we found that some other baseline characteristics of patients may be related to the clinical outcomes in the study as well. Results from MacDonald’s trial ^14^ reported no difference in HF hospitalization between the CA and MT groups. In the ARC-HF trial, ^15^ all-cause mortality was higher in the AF ablation group than in the MT group. Compared to the other trials, ^6 , 16 , 17^ the duration of AF in patients in the MacDonald’s and ARC-HF trials was significantly longer, exceeding 40 months. It suggested that patients with prolonged duration of AF may have led to a lower benefit from CA. Thus, early CA for AF in patients with impaired systolic function could be crucial in improving clinical outcomes. Most participants in the included trials were with persistent AF, ^14 - 18^ while the CASTLE-AF ^5^ and RAFT-AF ^6^ trials enrolled a subset of patients with paroxysmal AF. In both trials, the increase in LVEF associated with the ablation group was greater than in the other trials that included only persistent HF patients. This indicated that HF patients with paroxysmal AF may be better candidates for ablation. However, the AATAC trial, ^16^ enrolling HF patients with persistent AF, described the most significant reduction in all-cause mortality associated with ablation among the included trials. A possible explanation of the results could be that the MT group in the AATAC trial was treated with amiodarone to control the rhythm. Side effects of amiodarone might increase adverse outcomes in the drug group.

Moreover, we also noted that the left atrial diameter of patients in the ablation group of the ARC-HF ^15^ and AMICA ^18^ trials was larger than in the other studies, ^5 , 6 , 16 , 17^ reaching 50 mm. Pooled results from both trials reported that all-cause mortality in the CA group of both trials was higher than in the MT group. A larger left atrial diameter may be associated with more severe cardiac dysfunction and a greater burden of AF. It further suggested that patients with severe HF might benefit less from AF ablation. In brief, the effects of AF ablation vary in HF patient groups with different characteristics, so the selection of HF patients to undergo CA should be considered cautiously. More clinical controlled trials are needed to explore the stratification criteria and identify potential HF patient groups who could benefit more from AF ablation.

### Study limitations

The limitations include: First, this current study was limited by inconsistent eligibility criteria for each trial. The differences may result in heterogeneity when combining the data of these trials. Second, the RAFT-AF trial provided the primary outcome with a composite endpoint, which might impact the analysis results. In addition, the subgroup analysis was based on the baseline mean LVEF of patients in each study. The average LVEF could not represent the left ventricular function of each patient in studies.

Nevertheless, our work provided new ideas for future study. Moreover, due to the problems of the study itself, clinicians and patients were not blind to the treatment process. The subjective will of clinicians and patients may affect the outcomes.

## Conclusions

To the best of our knowledge, this is the first time to evaluate the efficacy of CA in AF patients with LVEF ≤45% in any of the meta-analyses published to date. Our study demonstrated that compared to MT, CA for AF in patients with HF was associated with a lower risk of all-cause mortality, hospitalization for HF, and recurrence of AF, and more significant improvements in LVEF and quality of life. Furthermore, the sub-analysis revealed that patients with milder left ventricular dysfunction could benefit more from AF ablation than patients with more severe disease. More research is needed to explore the characteristics of AF patients with HF that could benefit from CA.
